# The relationship between the gut microbiome and host gene expression: a review

**DOI:** 10.1007/s00439-020-02237-0

**Published:** 2020-11-22

**Authors:** Robert G. Nichols, Emily R. Davenport

**Affiliations:** 1 Department of Biology, The Pennsylvania State University, University Park, PA 16802 USA; 2 Huck Institutes of the Life Sciences, The Pennsylvania State University, University Park, PA 16802 USA

## Abstract

Despite the growing knowledge surrounding host–microbiome interactions, we are just beginning to understand how the gut microbiome influences—and is influenced by—host gene expression. Here, we review recent literature that intersects these two fields, summarizing themes across studies. Work in model organisms, human biopsies, and cell culture demonstrate that the gut microbiome is an important regulator of several host pathways relevant for disease, including immune development and energy metabolism, and vice versa. The gut microbiome remodels host chromatin, causes differential splicing, alters the epigenetic landscape, and directly interrupts host signaling cascades. Emerging techniques like single-cell RNA sequencing and organoid generation have the potential to refine our understanding of the relationship between the gut microbiome and host gene expression in the future. By intersecting microbiome and host gene expression, we gain a window into the physiological processes important for fostering the extensive cross-kingdom interactions and ultimately our health.

**Electronic supplementary material:**

The online version of this article (10.1007/s00439-020-02237-0) contains supplementary material, which is available to authorized users.

## Introduction

The human body plays host to large numbers of bacteria, fungi and other microorganisms—commonly referred to as the microbiota (Shreiner et al. 2015). These microbes are important to our health. They assist in establishing host immunity (Kamada et al. 2013), strengthen the gut barrier (Leclercq et al. 2014), and provide beneficial metabolites (Lukovac et al. 2014). Consequently, the microbiota is associated with a large number of complex diseases in humans, including inflammatory bowel disease (IBD) (Aleksandrova et al. 2017), cardiovascular disease (CVD) (Tang et al. 2017), and colon cancer (Nelson and Chia 2019). Whether shifts in the microbiota lead to or are the cause of disease remains largely unknown. Although, it has been demonstrated that the microbiota play causal roles in several diseases, including obesity (Ridaura et al. 2014; Turnbaugh et al. 2006) and diabetes (Wen et al. 2008). Given the importance for our health, it is necessary to characterize the physiological relationships between the microbiota and human host to understand disease etiology and design therapeutics involving the microbiome.

There is much to be learned about human–microbiome interactions by studying the genetic components of each. The human genome contains approximately 20,000 protein coding genes (Salzberg 2018). These genes are regulated in a tissue-specific manner by both intrinsic host factors as well as sensing environmental cues. Collectively, the microbial genomes within each of our microbiomes contain an estimated 100 times the gene content as our own genome (Nelson et al. 2010). This genetic material is sometimes referred to as our ‘second genome’, as the coding potential of the microbiome greatly expands upon the coding potential our own genome (Grice and Segre 2012). For example, genes unique to the microbiota create metabolites required by the human host (such as vitamin B12, biotin and folic acid) (Hooper et al. 2002) and allow for microbial survival (such as adhesion factors and transport systems) (Reidl et al. 2009). Understanding how the gene products of the host and microbiome interact can offer clues into what physiological processes are necessary for maintaining these complex cross-kingdom relationships.

In this review, we offer insights about the physiology of host–microbiota relationships gained by studying host gene expression jointly with the microbiome, specifically in the gut. We briefly describe the microbiome and how it is studied, synthesize themes gained through studies performed in powerful model systems and in humans, and highlight potential mechanisms through which host transcription–microbiota cross-talk occurs. Finally, we propose future directions, both experimental and analytical, that will further our understanding of how host transcription and the microbiome interact.

## The human gut microbiome: a primer

The relationship between the host and the gut microbiome starts at birth when the microbiome of the newborn is seeded. Delivery mode (cesarean section or vaginal delivery) plays an important role in the establishment of the microbiome (Bäckhed et al. 2015; Dominguez-Bello et al. 2010; Montoya-Williams et al. 2018; Papathoma et al. 2016). Proper nutrition and the transition from breast feeding to more solid foods results in maturation of the infant microbiome (Bäckhed et al. 2015). The establishment of the microbiome in neonates works in concert with the establishment of innate mucosal immunity, but can be disrupted by early use of antibiotics (Russell et al. 2012). The composition of the microbiome rapidly diversifies up to the age of three, steadily increases until around the age of 40, and then remains fairly stable (de la Cuesta-Zuluaga et al. 2019; Yadav et al. 2016; Yatsunenko et al. 2012). Though highly unique between individuals, the microbiome is relatively resistant to long-term changes (Bäckhed et al. 2012). The microbiome will typically return to a state of equilibrium after a stress like a dietary change, a short term adult antibiotic treatment, or an acute invasion by a pathogenic bacterium (Bäckhed et al. 2012). However, short-term modulations of the gut microbiome can interrupt normal metabolite production (Yoon and Yoon 2018). This in turn may cause changes in host gene expression that could lead to more long-lasting effects in the host.

To investigate the microbiome, researchers employ a plethora of techniques, many involving next-generation sequencing technology. To explore the taxonomic makeup of the gut microbiome, sequencing a phylogenetic marker gene is easy and efficient. Most often the 16S rRNA gene is assayed, as it is universally present in archaea and bacteria. The gene contains alternating segments of high and low conservation, which can be used for PCR priming and taxonomic identification, respectively (Davidson and Epperson 2018).

To explore microbial genomes and functional capacity of the gut microbiome, shotgun metagenomics is used. This involves sequencing the total DNA composition of a sample. Several aspects of the microbial community can be assayed using metagenomics, including characterizing taxonomic composition [through programs like Kraken (Wood and Salzberg 2014) and MetaPhlAn2 (Truong et al. 2015)], functional capabilities [through the program HUMAnN2 (Franzosa et al. 2018)], and assembling individual microbial genomes [metagenome assembled genomes, or MAGs (Albertsen et al. 2013; Allen and Banfield 2005; Nielsen et al. 2014; Parks et al. 2017)]. While metagenomics has the potential to reveal additional functional information over phylogenetic marker gene sequencing, it is expensive and can be cost prohibitive in samples with a high host to bacterial biomass ratio.

Finally, to explore functional activity of the gut microbiome, RNA within a microbiome can be sequenced via RNA-seq (referred to as metatranscriptomics). Typically, researchers deplete ribosomal and transfer RNA experimentally to enrich for bacterial transcripts prior to sequencing, as ribosomal and transfer RNA make up approximately 95–97% of total RNA in a bacterial cell (Rosenow et al. 2001). Metatranscriptomics investigates bacterial gene expression (transcription) changes between conditions or individuals [analyzed with the program SAMSA2, for example (Westreich et al. 2018)]. 16S rRNA gene sequencing, metagenomics, and metatranscriptomic sequencing can be coupled with host transcriptomics to investigate how different aspects of the gut microbiome (composition, functional capacity, or functional activity) affects host gene expression and vice versa.

## Relationship between microbiome and gene expression in model organisms

**Fig. 1:**
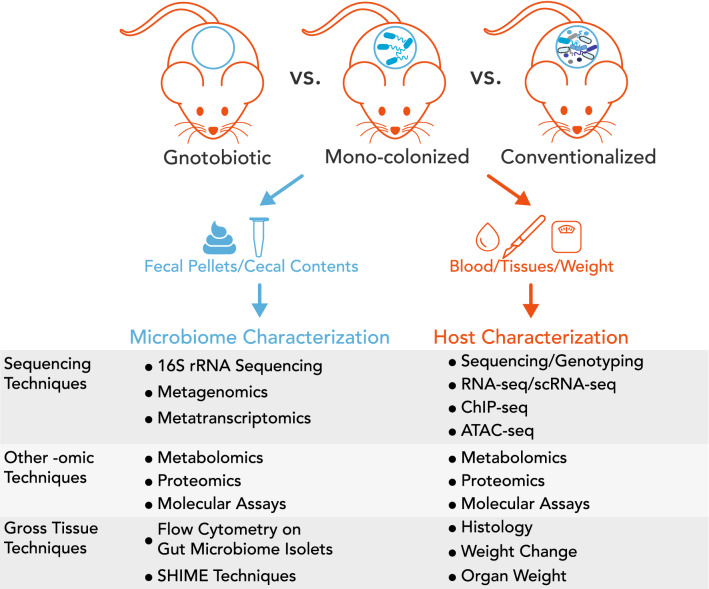
Using mice as a model organism. Mice are popular model organism for characterizing gut microbiome–host relationships. Researchers compare gnotobiotic (germ-free), mono-colonized, and/or conventionalized/wild-type mice to determine the role of the microbiome in their phenotype of interest. Orange elements represent host-derived tissues and techniques. Blue elements represent microbiome-derived samples and techniques. Both microbiome-derived and host-derived techniques can be categorized in three groups; sequencing techniques, other -omic techniques and gross tissue techniques. SHIME stands for the Simulator of the Human Intestinal Microbial Ecosystem (Molly et al. 1993)

A powerful way to investigate both the microbiome and host transcriptomics is with model organisms, such as mice (Fig. 1), zebrafish, *C. elegans* or *Drosophila melanogaster*. With model organisms, researchers have control over the environment, complete control over diet, and, importantly, the ability to study all tissues. These aspects are either impossible or extremely difficult to do with human subjects. Organisms bred without a microbiome (e.g., germ-free mice) can be used to identify gut microbiome-mediated effects in the host organism. For example, if the effects of a treatment are lost in germ-free mice when compared to wild-type or conventionalized mice, then it can be inferred that gut microbiome plays a role in the specific effect. While powerful, there are caveats to consider when using germ-free mice. With no microbiome present, there is much thinner mucous layer in the gastrointestinal (GI) tract of a germ-free mouse compared to a conventional mouse (Miyakawa et al. 1971). Barrier function is worse, and there is likely innate inflammation present in the gut (Miyakawa et al. 1971). Germ-free mice also have reduced metabolic rates and enlarged ceca (Miyakawa et al. 1971). These innate functional differences must be noted when drawing conclusions with germ-free mice.

Regardless, germ-free mouse experiments demonstrate that the microbiome plays a role in regulating host gene expression. For example, a murine model with a tissue-specific deletion of histone deacetylase 3 (*HDAC3*) results in the dysregulation of intestinal epithelial cell gene regulation dependent on the microbiome (Alenghant et al. 2013). Specifically, an intestinal epithelial cell specific *HDAC3* knockout mouse line was bred (HDAC3^ΔIEC^) to investigate inflammatory bowel disease (IBD) progression. Conventionalized HDAC3^ΔIEC^ mice show dysregulated host gene expression and disrupted homeostasis. Conversely, this dysregulation is not observed in a germ-free HDAC3^ΔIEC^ mouse model, demonstrating that the effects were mediated by the microbiome. These results highlight one mechanism by which the microbiome is sensed and affects gene expression in the host: by influencing the expression of a host epigenetic modifying enzyme.

Modulation of the microbiome (either by probiotics or creating germ free mice) can have dramatic effects on host gene expression. In a study focusing on 303 xenobiotic processing genes expressed in the intestine, 116 genes were significantly differentially expressed between mice raised conventionally vs. germ-free (Fu et al. 2017). These genes include Phase I enzymes, Phase II enzymes, transporters, and transcription factors. Additionally, expression of drug-metabolizing enzymes in the liver are significantly altered between groups of mice raised conventionally, conventionally + probiotics (VSL3), germ-free, and germ-free + probiotics (Selwyn et al. 2016). These studies demonstrate the causal role the microbiome plays in altering host gene expression across the body.

Host energy metabolism is affected by the microbiome, as evidenced by changes in host gene expression. Short-chain fatty acids (SCFAs) are small molecules made solely by the microbiome via microbial fermentation. In animal guts, these molecules are generated by the breakdown of host-indigestible foods and serve as a major energy source for the host. For example, an abundant SCFA of major health importance is butyrate, which colonocytes use as an energy source (Donohoe et al. 2011). In germ-free mice, the TCA cycle is dysregulated in colonocytes, due to the lack of butyrate (Donohoe et al. 2011). However, upon supplementation of butyrate, isolated colonocytes from a germ-free mouse dramatically increased mitochondrial respiration. Additionally, the microbiome influences host hepatic lipogenesis in wild-type mice. Increased monosaccharide absorption in the mouse gut suppresses the expression of fasting-induced adipocyte factor (*FAIF*), which results in the deposit of triglycerides in adipocytes (Bäckhed et al. 2004). These are just two examples of how microbes in the gut regulate host energy metabolism, even in distant tissues.

Host immunity is also affected by the microbiome. In a mouse model comparing wild-type mice to mice lacking innate immunity (*Myd88* knockout model) in both conventionally raised and germ-free conditions, over half of the expressed genes in the GI tract were regulated by the microbiome (2844 of 5652 genes) (Larsson et al. 2012). Gene Ontology (GO) analysis, showed the top differentially expressed genes were involved with host immune responses and host energy metabolism. Interestingly, the microbiomes involvement in both patterns of gene expression and dependency on MyD88 shifted along the GI tract. The composition, gene expression, and epigenetic profile of innate lymphoid cells (ILCs) are also shaped by the microbiome (Gury-BenAri et al. 2016). Specifically, single-cell RNA-seq, ATAC-seq, and iChIP-IVT on the intestinal lamina propria of conventional and antibiotic treated mice revealed hundreds of transcripts that were differentially regulated by the microbiome in several ILC clusters. These included genes related to cellular adhesion and interaction with the extracellular matrix, chemokine signaling, and MAPK signaling. In general, the transcriptomic profile of two ILC subsets (ILC1 and ILC2) shifted towards a third (ILC3). These results demonstrate the degree of plasticity host cells display in response to microbial stimuli and how that can affect downstream immune function. The relationship between the gut microbiome and host innate immunity has been extensively reviewed by Pott and Hornef (2012), Honda and Littman (2016), and Shi et al. (2017).

While we highlight mouse models above, many additional organisms serve as powerful laboratory systems to reveal insights into the relationship between the microbiome and host transcription. Studies conducted in zebrafish (Murdoch and Rawls 2019), *C. elegans* (Dirksen et al. 2020; Yang et al. 2019), and *Drosophila melanogaster* (Broderick et al. 2014; Douglas 2018; Elya et al. 2016) for example, all have revealed the impact of the microbiome on immunity, metabolism, and developmental gene regulation.

## Gastric pathogens influence host transcription

Given the difficulties of studying human gene expression, far less is known about the relationship between the human microbiome and the human transcriptome. The area with the most research to date is the study of how gut pathogens influence host gene expression, either directly or indirectly through immune stimulation. One of the ways pathogens modulate host gene expression is indirectly through the activation of host microRNAs (miRNA), which are small RNA molecules (~ 20 nucleotides long) that repress transcribed mRNAs in human cells by targeting specific RNAs for degradation or inhibiting their translation (Maudet et al. 2014). Pathogenic bacteria like *Listeria monocytogenes* (Schnitger et al. 2011)*, Salmonella Typhimurium* (Schulte et al. 2011) and *Helicobacter pylori* (Zhang et al. 2008) all stimulate host miRNAs that dampen the immune response, repress apoptotic signals, and increase autophagy to avoid host clearance (Maudet et al. 2014).

In addition to activating host miRNAs, pathogenic bacterial species release specific virulence factors called effector proteins which can either bind to host proteins to inhibit host cellular pathways or can act as enhancers or repressors for host genes (Shames and Finlay 2012). Pathogenic bacteria like *Salmonella Typhimurium* utilize their specific effector proteins to repress host innate immunity, leading to a successful invasion (Hausmann and Hardt 2019). In cases of *Clostridium difficile* infection (CDI), the two major toxins produced (TcdA and TcdB) increase expression of vascular endothelial growth factor A (VEGF-A) in gut epithelial cells (Huang et al. 2019). VEGF-A promotes angiogenesis and vasodilation and is upregulated in cases of IBD (Danese et al. 2006). Vasodilation exacerbates inflammation associated with IBD and promotes CDI pathogenesis (Huang et al. 2019). The toxins associated with CDI also downregulate the expression of aquaporins (specifically AQP1) in a human intestinal microvascular endothelial cell line (Hui et al. 2018). Irregular aquaporin activity results in diarrhea, due to disrupted osmosis (Hui et al. 2018).

Gut pathogens affect the host gene expression in a cell-type specific manner, and new techniques like dual RNA-seq and single cell RNAseq (scRNA-seq) can be used to investigate that relationship. Dual RNA-seq involves simultaneously sequencing both the pathogenic bacteria and the afflicted host cell (Westermann et al. 2012). This technique allows researchers to see the exact interactions that are occurring between the pathogen and the host cell (Westermann et al. 2016). The second technique mentioned, scRNA-seq, is a modified RNA-seq technique that involves sorting individual cells followed by RNA-seq to characterize the transcriptome on a cell-by-cell basis. ScRNA-seq allows for detection of gene expression differences both within and between different cell-types and characterization of cell-type proportions in a sample.

Currently, most scRNA-seq studies involving some microbiome component investigate the mechanisms underlying viral infection of host cells. For example, scRNA-seq revealed that there are highly heterogeneous infectivity and host transcriptional responses in a primary fibroblast model infected with herpes simplex virus 1 (HSV-1) (Drayman et al. 2019), identifiable transcriptomic signatures of SARS-CoV-2 infection in patients with severe disease (Bost et al. 2020), and no discernable differences between lytic and latent human cytomegalovirus (HCMV) transcriptomes in CD14 + monocytes and CD34 + HPCs (Shnayder et al. 2018). Though bacteria have different pathogenesis than viruses, scRNA-seq could be used to show how different microbial species of the microbiome affect the composition and transcriptional profiles of different host cells.

While exciting, technical limitations exist for scRNA-seq on host cells as well as for using scRNA-seq on a microbiome sample. The first hurdle of scRNA-seq is that the equipment needed for the experiments are expensive (Liu and Trapnell 2016). Additionally, the procedures used to isolate cells and their respective RNA are complicated and could introduce bias based on the enzyme treatments used. ScRNA-seq methods are most criticized for their low capture rates, meaning that sparse transcripts may be missed. When applying scRNA-seq to the microbiome, the above issues are compounded with the fact that there are thousands of species of bacteria in our gut. There has not been a method developed for using scRNA-seq specially on a microbiome sample. However, one group took existing scRNA-seq methods that address multiple species (Butler et al. 2017), zero inflation issues (Van den Berge et al. 2018) and issues with technical noise (Kharchenko et al. 2014), tested them on simulated and manually curated 16S and metagenomic microbiome data, and compared the results with existing metagenomic analysis techniques (Calgaro et al. 2020). This group concluded that there was no perfect method but existing microbiome methods like DESeq2, edgeR and corncob preformed the best when analyzing the data. The prospect of combining scRNA-seq with the microbiome is attractive, new analytical techniques will need to be implemented to deal with the complexity of both the host and the microbiome.

## Relationship between the gut microbiome and host transcription in humans

**Fig. 2:**
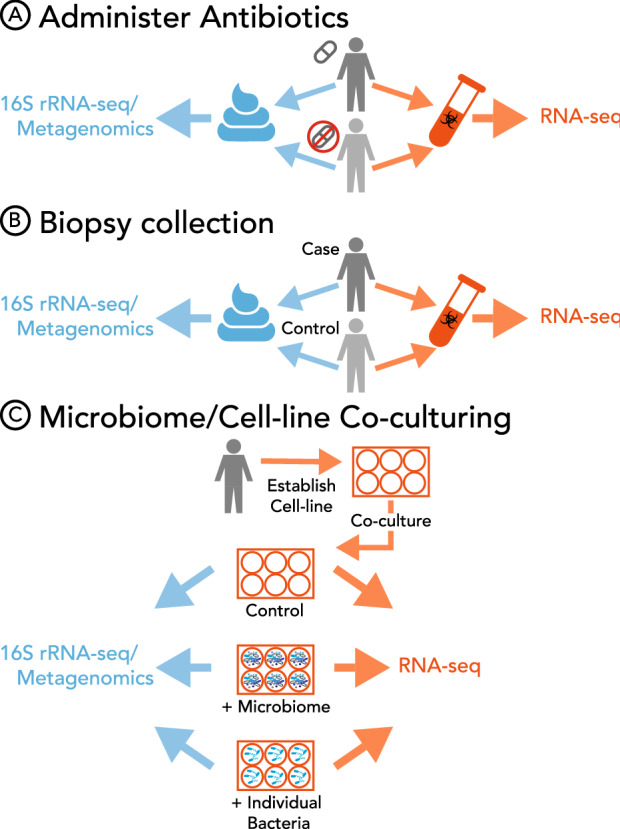
Techniques for examining microbiome—gene expression relationships in humans. Several different approaches are used to investigate the relationship between the microbiome and host transcriptome in humans. **a** Human subjects are split into two groups: one group is placed on antibiotics to temporally reduce the gut microbiome, while the other acts as a control. Researchers collect fecal and blood or tissue samples to study the microbiome and host transcriptome, respectively. **b** Tissue biopsies are collected during medical procedures from both inflamed and non-inflamed tissue, for example during colonoscopies. RNAseq data are collected from the host tissue, while the microbiome is characterized from either the associated mucosal layer or a separately collected fecal sample. **c** Human cell lines are established and co-cultured in the presence of a microbiome, individual bacterium, or vehicle control. Differences in gene expression between conditions can then be attributed to the microbiome

When investigating microbiome–host interactions in humans, researchers employ various approaches, such as collecting biopsies (taken during colonoscopies, gastric surgeries, or colonic surgeries), generating organoids, the gut on a chip model (Kim et al. 2016) or using human cell cultures (Fig. 2, Supplemental Table 1). Biopsies provide the most biologically faithful representation of host gene expression–microbiome relationships at the interface of the intestinal lumen, but suffer from the drawback that they are invasive to obtain and are difficult to maintain the features of ‘normal’ human tissue. Consequently, many profiled biopsies originate from diseased tissue, and do not offer insight into host–microbiome interactions in healthy states. In some instances, matching control samples are collected from patients with diseased or inflamed tissue (Häsler et al. 2017; Lloyd-Price et al. 2019). This matched study design allows direct comparison between “normal” and inflamed tissue, which can be a more powerful approach that takes into account host-specific factors like diet and genetics versus comparing case and control samples collected in different individuals.

One such study used biopsies taken from the terminal ileum and sigmoidal colon from healthy individuals and those with IBD (inflamed and healthy intestinal tissue) to study IBD pathophysiology in humans (Häsler et al. 2017). Specifically, when comparing inflamed and non-inflamed tissues in the same individual, 13 pathways were differentially expressed in host tissue, including repression of citric acid cycle, bile acid synthesis and fatty acid oxidation, and induction of tryptophan, glycine, serine, alanine and threonine metabolism. Additionally, ~ 90% of the genes were differentially expressed between healthy and IBD individuals correlated with microbial taxa present in the healthy state and had almost no correlation to the microbial taxa present in the IBD gut. This “uncoupling” of mucosal gene regulation is hypothesized to be an important component of the environmental-host axis underlying IBD etiology.

A second study, also examining IBD progression used biopsies taken from patients during routine colonoscopies with both normal and inflamed intestinal tissue (4–14 biopsies per patient) coupled with stool and blood samples (Lloyd-Price et al. 2019). Interestingly, antimicrobial genes, including *CXCL6*, *LCN2*, *DUOX2* and *SAA2,* showed increased expression in patients with IBD compared with controls. A separate study also saw increases in antimicrobial host genes in IBD [specifically those with ulcerative colitis (UC)] patients when compared to control (Bennet et al. 2018). The increase of antimicrobial genes in IBD patients could be one of the many reasons for the microbiome dysbiosis observed in the disease, due to the direct impact host antimicrobial genes would have on the members of the gut microbiome. This might also suggest that host gene expression plays a larger role in altering microbiome composition than the microbiome plays on altering host gene expression in cases of IBD. However, during outbreaks of pouchitis in patients who underwent ileal pouch-anal anastomosis as a treatment for IBD, the host transcriptome of ileal tissue drastically changed within the ileal pouch, while the taxonomic makeup of the microbiome remained stable between the ileal pouch and pre-pouch ileum (Morgan et al. 2015). This suggests that microbiome can act independently of host gene expression changes, and that the relationship between the two is highly dependent on disease context.

Biopsies are highly invasive and expensive, and alternative strategies exist for studying the relationship between the human microbiome and transcriptome. Cell cultures are cheap, relatively easy to maintain, and can be completely controlled by the researcher. To study how the host cells and microbes interact, co-culture techniques can be employed where intestinal cells are co-cultured with fecal isolates to simulate a host–microbiome environment (Richards et al. 2016). Although an imperfect proxy, colonocyte-microbiome co-culture experiments identify thousands of genes differentially regulated in the presence of a microbiome compared to control. The differentially expressed genes in the co-culture model are significantly enriched for genes identified as differentially expressed in murine colonic epithelial cells from conventional vs. germ-free mice, demonstrating the biological utility of the model.

## The microbiome and host gene expression: a two-way conversation

**Fig. 3:**
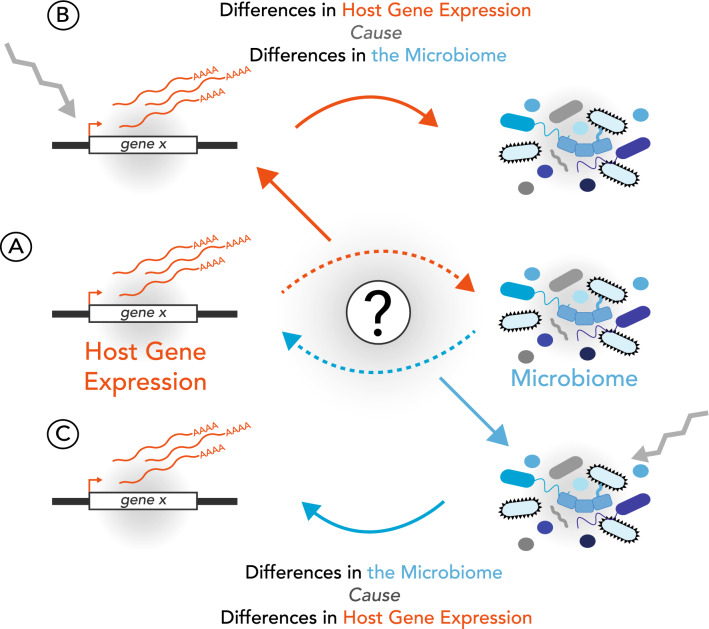
Direction of causality between microbiome and host gene expression associations. **a** When an association between the microbiome and host gene expression is identified, an open question is whether the microbiome is leading to the changes in gene expression or vice versa. **b** Here, differential expression of a host gene expression leads to changes in the microbiome. **c** Here, changes in the microbiome cause a change in host gene expression

It is clear from the many studies in model organisms and humans that there is an association between the gut microbiome and gene expression in the host. In many cases, it is unclear what the direction of causality is with these associations, however. Does a change in microbiome composition cause changes in host gene expression, or does a change in host gene expression change microbiome composition (Fig. 3)? Deciphering this relationship is important for understanding disease etiology and ultimately designing therapeutics that target the microbiome.

Comparing microbiome-containing to germ-free system is one way to assess whether the microbiome plays a causative role in regulating gene expression. This type of approach can be done by comparing either germ-free to conventional organisms (Bäckhed et al. 2004; Fu et al. 2017; Larsson et al. 2012; Sayin et al. 2013; Selwyn et al. 2016) or by comparing cell cultures co-cultured with a microbiome or un-inoculated media (Richards et al. 2016). For example, expression of intestinal Cytochrome P450 (Cyp) 3a sub-family and transporter genes are significantly decreased in germ-free compared to conventionalized mice (Fu et al. 2017). Lower expression of these genes drastically reduces the detoxification capability of the host. The decrease in expression of these genes occurs in the germ-free host, demonstrating the causal role the microbiome plays in regulating detoxification pathways in the host.

In population samples, where germ-free conditions are unavailable, Mendelian Randomization (MR) offers a tool to assess whether the microbiome leads to a phenotype in the host or vice versa (Wade and Hall 2020). For example, MR analysis revealed that the gut microbiome casually effects metabolic traits related to type 2 diabetes and obesity (Sanna et al. 2019). Although not applied to studies of gene expression and the microbiome yet, this would be a powerful framework to examine direction of causality between the host transcriptome and microbiome.

Conversely, genome-wide association studies (GWAS) of the gut microbiota provide evidence that gene expression likely influences the abundance of certain bacteria in the gut (Blekhman et al. 2015; Bonder et al. 2016; Davenport 2016; Goodrich et al. 2016; Goodrich et al. 2017; Turpin et al. 2016; Wang et al. 2017). In particular, taxa such as Christensenellaceae, *Akkermansia*, and *Bifidobacterium* are either heritable or associated with genetic variation in the human genome. As it is expected that an individual’s genome sequence will not change in the presence of the microbiome, these studies demonstrate that host genetics, likely via gene regulation, modulates aspects of the gut microbiome.

## Molecular mechanisms linking the microbiome to host gene expression

**Fig. 4:**
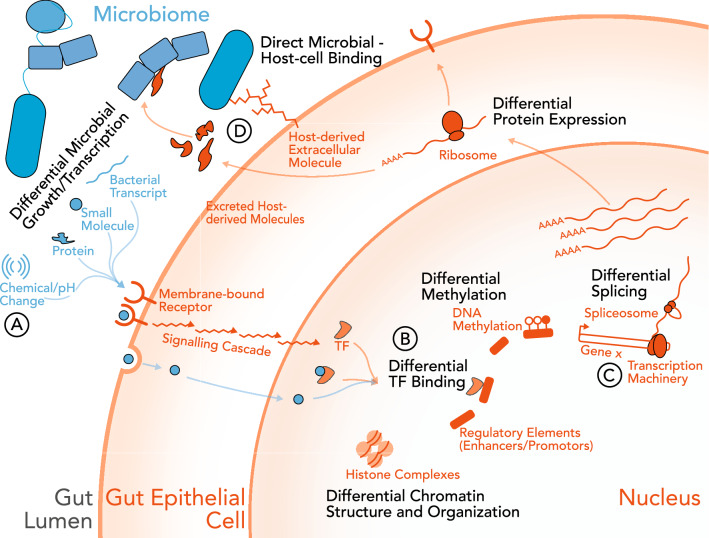
Potential mechanisms underlying host gene expression–microbiome associations Interactions between the microbiome and microbiome-derived molecules and host can occur in either cellular or extra-cellular compartments and involve a variety of processes. **a** Microbiome derived stimuli are recognized by host cells, either via interaction with extracellular receptors or entering the cell. Stimuli include microbiome-derived transcripts, small molecules, proteins and enzymes, or pH changes. **b** Microbiome-derived molecules cause differential transcription factor binding, methylation, or chromatin availability. These changes lead to differential expression of host genes. **c** Microbiome-derived molecules can also lead to changes in transcription and alternative splicing, resulting in different host gene products. Both **b** and **c** result in differential protein expression, that are then released back into the gut lumen. **d** Host-derived proteins can also modulate the bacteria present in the gut microbiome, causing differential microbial growth and transcription. Microbiome-derived molecules illustrated in blue. Host-derived molecules illustrated in orange

Many molecular mechanisms foster the cross-talk between the microbiome and host gene expression (Fig. 4). Transcription factors are host proteins that bind to DNA and regulate the transcription of genes. Elements of the microbiome bind directly to transcription factors (Davison et al. 2017; Krautkramer et al. 2017). In zebrafish, the microbiome suppresses the transcription factor hepatocyte nuclear factor 4A (*HNF4α*), preventing the regulation of host inflammatory pathways, potentially leading to an inflammatory state (Davison et al. 2017). Studies on the skin microbiome of mice showed that colonization of the skin microbiome regulates the expression of several key transcription factors (*Klf4*, *AP-1* and *SP-1*), albeit via an unknown mechanism (Meisel et al. 2018).

The human microbiome also affects epigenetic modifications like DNA methylation and histone acetylation (Krautkramer et al. 2017; Yu et al. 2015) (Fig. 4b). For example, germ-free mice have lower genome-wide DNA methylation in colonic tissue compared to conventionally raised animals (Yu et al. 2015). A fecal transplant, however, drastically increases global DNA methylation in previously germ-free mice. Additionally, short-chain fatty acids (SCFAs) influence histone acetylation (Krautkramer et al. 2017). Even a small change in diet can cause major shifts in SCFAs and other bacterial metabolite levels in the host (David et al. 2014). Taken together, small dietary changes can potentially have major effects on host histone post-translational modifications, which then could change host gene expression for a variety of genes.

The microbiome also remodels the chromatin in host intestinal epithelial cells (Fig. 4b), albeit with conflicting evidence. ATAC-seq in a colonic epithelial cell co-culture model demonstrates that specific microbes regulate chromatin accessibility and transcription factor binding in host tissues (Richards et al. 2019). In mouse models, the presence of a microbiome results in more highly accessible chromatin in intestinal epithelial cells as compared to a gnotobiotic mouse (Semenkovich et al. 2016). The authors even speculate that the intestinal epithelium could have evolved to have a chromatin structure that requires a microbiome to activate appropriately. However, a separate study showed there are not significant changes in chromatin accessibility in intestinal epithelial cells between wild-type mice and germ-free mice (Camp et al. 2014). Instead microbial regulation of host gene expression most likely came from different transcription factor binding regions in the available chromatin.

Conversely, in a diet-induced obesity mouse experiment, the microbiome remodels chromatin in colonic cells (Qin et al. 2018). This results in an increase of accessible HNF4α binding sites, subsequently leading to downregulation of genes near those sites. One important gene that gains an HNF4α-binding site and is downregulated is *Scd1* (Qin et al. 2018). *Scd1* is an important regulator of the amount of free floating fatty acids and is responsible for combining them into triglyceride storage (Miyazaki et al. 2000). Downregulation of *Scd1* has been seen in patients with nonalcoholic fatty liver disease (NAFLD) (Gornicka et al. 2011). Taken together it is possible that the microbiome in an obese state indirectly promotes the formation of NAFLD through modulation the structure of the chromatin in colonic cells, resulting in more binding sites for HNF4α.

Finally, the gut microbiome also has a role in the alternative splicing of host genes (Martínez-Montiel et al. 2016) (Fig. 4c). Products from *Pseudomonas sp* (FR901464) and *Streptomyces sp* (pladienolide) inhibit host splicing machinery (Fan et al. 2011). Additionally, 320 differential splicing events in intestinal tissue occurred between patients with IBD and healthy controls, after controlling for tissue type, inflammation status, and diagnosis. (Häsler et al. 2017). The alternative splicing events upregulated in IBD patients were mapped to the KEGG pathways for ‘bacterial invasion of epithelial cells’, ‘pathogenic *E. coli* infection’ and ‘allograft rejection’. It should be noted that there was only a weak correlation between host gene expression and the shared alternative splicing events. Research into the relationship between alternative splicing and the microbiome remains scarce and should be considered in future studies, considering the major role splicing plays in complex disease etiology (Li et al. 2016).

## Future directions

The complexity of the interactions between the microbes in the gut and the host is immense. Although recent studies in model organisms and humans demonstrate that trans-kingdom crosstalk occurs, there are still many avenues to explore to gain further insight into host–microbiome interactions. Many existing studies identify individual bacteria associated with host gene expression. While this can occur, other effects are likely driven by a variety of taxa, due to functional redundancy. There are thousands of bacterial species present in the gut microbiome. Many different species share genes and pathways to produce the same metabolites (Moya and Ferrer 2016). Therefore, it is important to understand how the functional capability of the gut microbiome changes, in addition to shifts in taxonomy (Blakeley-Ruiz et al. 2019; Schirmer et al. 2019). Bacterial metagenomics, metatranscriptomics, and metaproteomics can be used to investigate the functional potential of the gut microbiome. Future studies that combine metatranscriptomics, metaproteomics, and 16S sequencing with metabolomics and RNAseq of host tissue may be able to reveal more insights about how microbiome functions interact with host gene expression.

As briefly mentioned above, scRNA-seq of both the microbiome and host tissue has the potential to reveal how the microbiome associates with cell-type composition and cell-specific gene expression. For example, using scRNA-seq, the transcriptome of only the first layer of intestinal epithelial cells that have direct contact with the microbiome could be assayed. This would limit the noise in gene expression measurements collected from multiple cell types and instead focus analysis on only the interactions happening directly at the interface of the host–gut axis.

Additionally, organoid systems can be utilized to create a better in vitro model of the human gut*.* Organoid systems for human colon and small intestinal tissue contain crypts and villi, which more accurately model an intestinal system versus cells adherent to a dish (Sato et al. 2011). Recently, intestinal organoids were used to model *Clostridium difficile* infections (CDI) (Leslie et al. 2015). The CDI organoid model more closely represented in vivo CDI when compared to CDI modeled with a cell culture (Leslie et al. 2015). The next step for these types of studies would be to move on to organoid studies and co-culture organoids with microbiome isolates. Recent methods involving microinjection could be employed to recapitulate the gut microbiome inside the organoids to provide researchers with a better model to study host–gut interactions (Williamson et al. 2018). Additionally, organoids created from patients with Crohn’s disease and healthy controls showed that the ex vivo organoid model could recapitulate the DNA methylation profiles seen in both host tissue (Howell et al. 2018). Organoid models have also been used to investigate intestinal cytokine secretion, independent of host immune functionality, to show that cytokine secretion is dependent on differentiation state (Lyons et al. 2018). Therefore, while we cannot make germ-free humans or knockout humans (like we can with mice), researchers can utilize 3D organoids with and without the microbiome instead.

Finally, novel computational methods need to be developed that integrate multiple highly complex datasets together to reveal biological insight, such as gene expression and taxonomic composition. Each type of high dimensional data has its own quirks, such as sparsity, compositionality, or overdispersion (Anders et al. 2010; Tsilimigras and Fodor 2016). Designing tools that specifically take these issues into account will allow researchers to identify novel and complex associations between the gut microbiome and host gene expression.

The study of the relationship between the microbiome and host gene expression has already yielded great insights into the physiological mechanisms that link a host with the microbes that live within it. With the exponential growth of sequencing techniques and technologies, new cell culture techniques, the growing popularity of scRNA-seq, and the advent of more advanced statistical methods, we are currently poised to gain even greater insight into the trans-kingdom relationship we have with our microbiome.

## Electronic supplementary material

Below is the link to the electronic supplementary material.

Supplementary file1 (XLSX 14 KB)
